# Inflammation-Mediated Immune Imbalance in the Pathogenesis of Diabetic Cataracts

**DOI:** 10.3390/biomedicines14020372

**Published:** 2026-02-05

**Authors:** Nan Gao, Xiteng Chen, Guijia Wu, Zhenyu Kou, Jun Yang, Yuanfeng Jiang, Ruihua Wei, Fang Tian

**Affiliations:** Tianjin Key Laboratory of Retinal Functions and Diseases, Tianjin Branch of National Clinical Research Center for Ocular Disease, Eye Institute and School of Optometry, Tianjin Medical University Eye Hospital, 251 Fukang Road, Nankai District, Tianjin 300384, China; gn19970000@126.com (N.G.); chxt88@126.com (X.C.); 17638028275@163.com (G.W.); kzy01212@163.com (Z.K.); tj_yangjun@2008.sina.com (J.Y.); frankjyf@126.com (Y.J.)

**Keywords:** diabetic cataract, type 2 diabetes, immune imbalance, RNA sequencing, single-cell RNA sequencing

## Abstract

**Background:** Diabetes increases the risk of cataract formation fivefold. Immune-mediated inflammation has been reported to play a role in this process; however, whether alterations in the immune landscape are involved remains unknown. Therefore, we conducted a multi-omics analysis to evaluate the impact of immune inflammation on the lens. **Methods:** Bulk RNA sequencing was performed on peripheral blood mononuclear cells (PBMCs) from diabetic patients and lens tissues from diabetic rats. Single-cell RNA sequencing was utilized to characterize intercellular interactions. Key gene and protein expressions were validated via laboratory assays. **Results:** An integrated RNA-seq analysis revealed a disruption of the blood–aqueous barrier integrity in the diabetic group, enhanced monocyte migration and adhesion, increased differentiation from classical to non-classical monocytes, and the upregulation of TNF and IFN-γ signaling pathways. The transcriptomic profiling of rat lenses revealed an increased proportion of monocytes and the activation of apoptotic pathways in lens epithelial cells. Immunohistochemistry and immunofluorescence staining demonstrated elevated caspase-3 and IL-6 levels in lens epithelial cells and increased immune cell infiltration in the diabetic group. The qRT-PCR and ELISA confirmed elevated levels of the pro-inflammatory cytokines IL-6 and IFN-γ, alongside reduced anti-inflammatory cytokine IL-10 in the peripheral blood and aqueous humor of diabetic patients. **Conclusions:** Diabetes alters the peripheral immune microenvironment and disrupts the blood–aqueous barrier, promoting intraocular inflammation and lens epithelial cell apoptosis, thereby accelerating cataract development.

## 1. Introduction

Type 2 diabetes is a risk factor for cataract formation. Inflammation, oxidative stress, and elevated levels of advanced glycation end-products induced by diabetes contribute to cataract development [1,2]. Patients with diabetes mellitus have an up to fivefold higher risk of early cataract onset relative to non-diabetic individuals, particularly in relation to cortical and posterior subcapsular cataracts [2]. According to the most recent International Diabetes Federation report, the global diabetic population is projected to increase from 463 million in 2019 to 700 million by 2045 [3]. This rising prevalence of type 2 diabetes will exacerbate the visual impairment and healthcare burden associated with cataracts. Therefore, it is important to clarify the role of type 2 diabetes in cataract pathogenesis.

Although the eye is an immune-privileged site, immune cells can still be recruited and cause disruptions after a pathogenic insult [4,5]. It has been proven that the immune response plays an important role in multiple ocular diseases, including cataracts [6]. A recent study demonstrated immune cell infiltration into the lens capsule in a mouse model of uveitis, suggesting a mechanism by which uveitis may accelerate cataract progression [4]. Notably, a series of studies by DeDreu and colleagues indicated that immune cells can reach the lens surface through zonular fibers, an observation established both during lens development and in response to corneal injury. This pathway may represent a source of immune cells for the lens [5,7]. Other studies have also highlighted the intrinsic connection between immune responses and lens pathologies [8,9]. However, there remains no direct evidence linking immune activity to the pathogenesis of diabetic cataracts.

Advances in modern research technologies have driven omics studies toward greater quantification and high-throughput capabilities [10]. The application and analysis of omics data provide deeper insights into disease mechanisms and pathological processes [11,12]. Moreover, with the advancement of RNA sequencing toward single-cell resolutions, the integration of a single-cell analysis with bulk RNA sequencing data enables the more precise identification of cellular and signaling pathway alterations during disease progression, thereby enhancing the accuracy of bioinformatics analyses.

In this study, we performed high-throughput sequencing of peripheral blood mononuclear cells (PBMCs) from patients with type 2 diabetes mellitus and integrated single-cell transcriptome data. Through bioinformatics analyses, we explored cytokine alterations and mechanisms of immune imbalance that may contribute to the pathological process of diabetic cataracts from the perspectives of gene expression and cellular regulation.

## 2. Materials and Methods

### 2.1. PBMC Collection and Differential Analysis

Blood samples were collected from 24 patients who underwent cataract surgery at Tianjin Medical University Eye Hospital. The cohort included 16 patients with diabetes mellitus (DM group) and 8 patients without diabetes mellitus (NDM group). The inclusion criteria were as follows: (1) in the DM group only—a confirmed diagnosis of type 2 diabetes by an internist; (2) no history of ocular diseases or ocular surgery; and (3) the absence of other systemic metabolic disorders, corticosteroid use, hepatic or renal disease, malignant tumors, or immunological diseases. This study adhered to the principles of the Declaration of Helsinki. Written informed consent was obtained from all participants, and the study protocol was approved by the Ethics Committee of Tianjin Medical University Eye Hospital (Ethics No. 2023KY(L)-21). The transcriptome sequencing of PBMCs was performed using the DNBSEQ-T7 HiSeq platform (MGI Tech, Shenzhen, China). Differentially expressed genes (DEGs) between the DM and NDM groups were identified using the R package DESeq2 (version 1.30.1) [13]. The cutoff criteria for DEGs were set at |log_2_ fold change| > 0.5 and *p* < 0.05.

### 2.2. Weighted Gene Co-Expression Network Analysis

A co-expression network was constructed using the R package WGCNA (version 1.72-5) [14] with standardized gene expression data, and outlier samples were removed based on a clustering analysis. A similarity matrix was generated by calculating pairwise correlation coefficients for genes. A soft threshold was applied to transform the similarity matrix into an adjacency matrix, ensuring a scale-free network topology. A topological overlap matrix was then calculated to evaluate the average connectivity between genes. Genes with similar expression profiles were grouped into modules using a dynamic tree-cutting algorithm; each module contained at least 30 genes. Module dissimilarity was calculated, and hierarchical clustering was used to merge modules with a dissimilarity below 0.25. Modules were visualized in distinct colors; genes not assigned to any module were placed in the gray module. Module eigengenes were used to represent the overall expression patterns of individual modules and to assess associations between modules and phenotypes. Modules with the highest absolute correlation coefficients were considered key modules and subjected to further analysis. Module membership was defined as the correlation between a gene’s expression value and the eigengene of its module, whereas gene significance represented the correlation between gene expression values and the phenotype.

### 2.3. Immune Infiltration Analysis and Functional Enrichment

CIBERSORTx (https://cibersortx.stanford.edu/, accessed on 6 March 2024) [15] was utilized to establish an immune cell reference transcriptome library and conduct an immune infiltration analysis, enabling a comparison of immune cell subpopulations between the two cohorts. Gene Ontology (GO) and Kyoto Encyclopedia of Genes and Genomes (KEGG) functional enrichment analyses of overlapping genes were performed using the clusterProfiler package (version 3.18.1).

### 2.4. Single-Cell Transcriptome Analysis of PBMCs and Lenses

The single-cell transcriptome analysis was conducted using the GSE244515 [16] (GSM7818506, GSM7818507, GSM7818508, GSM7818516, GSM7818517, GSM7818518) and GSE199013 [17] datasets from the Gene Expression Omnibus database. A canonical correlation analysis was performed to identify mutual nearest neighbors [18]. Cells expressing fewer than 200 or more than 2500 genes, or with mitochondrial RNA content exceeding 5%, were excluded; the remaining data were normalized. Variable genes were identified using the “vst” method and then subjected to dimensionality reduction with t-distributed stochastic neighbor embedding (t-SNE) based on principal components selected using Jackstraw analysis (*p* < 0.05) (Based on Seurat package, version 4.0.2). Distinct cell clusters were identified according to DEGs at a resolution of 1. For PBMC single-cell transcriptome data, cells were annotated using the MonacoImmuneData database in singleR (version 1.4.1), along with established cell markers. DEGs between the two groups were identified in each cell type using the Wilcoxon test, followed by an enrichment analysis. Ligand–receptor interactions between PBMCs were assessed using the CellChat package (version 1.4.0); differential receptor interactions between the diabetes and non-diabetes groups were compared. To investigate developmental relationships between PBMC subtypes, a cell trajectory analysis was performed using the Slingshot algorithm. Associations between gene expression and cell development were evaluated with tradeSeq (version 1.16.0). For single-cell transcriptome lens data, cells were annotated using marker genes for lens epithelial cells and fiber cells, and the expression distributions of selected immune cell markers were examined.

### 2.5. Diabetic Cataract Animal Model

This study utilized Sprague Dawley (SD) rats purchased from Vital River Laboratory Animal Technology Co., Ltd. (Beijing, China). All animals were housed in a specific pathogen-free facility at Tianjin Medical University Eye Hospital. The housing conditions comprised a 12-h light/dark cycle, with a relative humidity of 40–70% and a constant temperature of 26 °C. Food and water were provided ad libitum. Diabetes was induced in rats via an intraperitoneal injection of streptozotocin (STZ) (Sigma-Aldrich, St. Louis, MO, USA). Prior to treatment, rats were fasted overnight, and streptozotocin was freshly prepared in a sodium citrate buffer at a 1:100 ratio. Rats received a single injection of STZ at a dose of 60 mg/kg. Blood glucose levels were measured on the third day post-injection. If the blood glucose level did not increase, a supplementary dose of 55 mg/kg STZ was administered. The control group received sodium citrate buffer injections. Rats with glucose levels exceeding 14 mmol/L were retested, and those with persistently elevated values were classified as diabetic. During this study, random tail tip blood glucose measurements were conducted every three days. Insulin therapy was administered when blood glucose levels persistently exceeded 25 mmol/L. Rats that exhibited a deteriorated health status and were unable to tolerate the experimental procedures were euthanized as a humane endpoint. The animals were deeply anesthetized via isoflurane inhalation followed by euthanasia via cervical dislocation. Death was confirmed by the absence of spontaneous respiration and the loss of a pedal reflex in response to a toe pinch. Lens samples were collected from three diabetic rats that developed cataracts and three control rats. The sample size was determined based on previous literature [19,20,21]. No mortality or other adverse events were observed throughout the study. All animal experiments were approved by the Tianjin Medical University Eye Hospital Animal Care and Use Committee (Ethics No. TJYY2023120169).

### 2.6. Rat Lens RNA Sequencing and Bioinformatics Analysis

The total RNA was extracted from rat lenses and sequenced using the Illumina NovaSeq 6000 platform (Illumina, San Diego, CA, USA). Quality control procedures for Q20 and Q30 metrics were performed on raw sequencing data using FastQC (version 0.11.5), followed by the alignment of clean reads to the reference genome GCF_015227675.2. A differential expression analysis was conducted using DESeq2 (version 1.30.1); genes meeting the criteria of |log_2_ fold change| > 0.5 and *p* < 0.05 were considered DEGs. GO and KEGG pathway enrichment analyses were performed on the identified DEGs. Immune cell infiltration was assessed using CIBERSORTx (https://cibersortx.stanford.edu/, accessed on 26 September 2024) [15].

### 2.7. Immunohistochemical Staining

During cataract phacoemulsification surgery, anterior lens capsules were carefully excised from six patients and fixed at room temperature for 3 h in 4% paraformaldehyde. The samples were then washed three times with phosphate-buffered saline (PBS). Appropriately sized tissue fragments were placed in embedding cassettes and subjected to a graded dehydration protocol: 75% ethanol for 1.5 h, 95% ethanol for 1.5 h, 95% ethanol for 1 h, absolute ethanol for 1.5 h, absolute ethanol for 1 h, xylene 1 for 30 min, and xylene 2 for 30 min. After dehydration, the tissues were infiltrated with molten paraffin wax. The wax-embedded specimens were transferred into molds, filled with molten paraffin, and allowed to cool and solidify to complete the embedding process. The paraffin blocks were pre-cooled at 4 °C, fixed onto a microtome specimen holder, and sectioned at a thickness of 4 μm. The sections were floated in 40 °C water to flatten and then baked at 60 °C for 30 min. Paraffin was removed by sequentially immersing the sections in xylene 1, 2, and 3 for 5 min each. The samples were then rehydrated in absolute ethanol, 90% ethanol, 80% ethanol, and 70% ethanol for 5 min each; this was followed by two washes in distilled water for 5 min each. For antigen retrieval, the slides were heated in an antigen retrieval solution (10 mM Tris–ethylenediaminetetraacetic acid, pH 9.0) at 95 °C for 10 min and then cooled to room temperature. They were then washed three times with PBS for 5 min each and incubated in 3% hydrogen peroxide at room temperature for 10 min, followed by three additional washes with PBS for 5 min each. Blocking was performed for 30 min using 1% bovine serum albumin (BSA; prepared by mixing 0.1% Tween-20 and 5% BSA at a 4:1 ratio). The primary antibody (1:200 dilution; AF6311, DF16080, Affinity, Changzhou, China) was diluted in 1% BSA and applied to the slides for 1 h at room temperature. After three PBS washes, the horseradish peroxidase-conjugated secondary antibody (1:200 dilution; S0001, Affinity, Changzhou, China) was added and incubated at room temperature for 30 min. Three additional washes with PBS were performed; 100 μL of a diaminobenzidine solution was applied, and staining was monitored under a microscope for 3–10 min until an optimal intensity was achieved. The reaction was then terminated, and the sections were rinsed with PBS. The slides were then counterstained with 100 μL of hematoxylin for 1–3 min at room temperature until the nuclei appeared blue; this was followed by a rinse with PBS to restore the blue coloration. The sections were sequentially dehydrated in 70% ethanol for 1 min, 80% ethanol for 1 min, 95% ethanol for 2 min, absolute ethanol for 4 min, xylene 1 for 3 min, and xylene 2 for 3 min. A neutral mounting medium was applied, and coverslips were placed on the sections, which were then dried before microscopic imaging. Finally, the average staining intensity and the proportion of positive cells were quantified using Fiji ImageJ software (version 1.1.8.0).

### 2.8. Immunofluorescence Staining

During the cataract phacoemulsification surgery, anterior lens capsules from patients (*n* = 6) were carefully excised and fixed in 4% paraformaldehyde for 2 h at room temperature. The capsules were transferred to a 48-well plate, washed three times with PBS, and blocked for 1 h with 1% BSA (prepared by mixing 0.1% Tween-20 and 5% BSA at a 4:1 ratio). The primary antibody against interleukin (IL)-6 (1:200, DF6087, Affinity, Changzhou, China) was diluted in 1% BSA and applied to the slides overnight at 4 °C. After three washes with PBS, the capsules were incubated with a fluorescently labeled secondary antibody (1:200, S0006, Affinity, Changzhou, China) for 30 min at room temperature. Nuclei were counterstained with 4′,6-diamidino-2-phenylindole (DAPI) for 1 min at room temperature and then washed three times with PBS. Specimens were flat-mounted on slides, and images were captured using a fluorescence microscope (Olympus, Tokyo, Japan). The mean immunofluorescence intensity was quantified using Fiji ImageJ software (version 1.1.8.0).

### 2.9. Quantitative Reverse Transcription Polymerase Chain Reaction (qRT-PCR)

Peripheral blood samples were collected from the simple cataract control group (*n* = 8) and the diabetic cataract group (*n* = 8). mRNA expression levels of the immunomodulatory factors interferon (IFN)-γ, IL-6, and IL-10 were assessed via qRT-PCRs using the 7900HT Fast Real-Time PCR System. The total RNA was extracted with the TRIzol reagent (Invitrogen, Carlsbad, CA, USA), in accordance with the manufacturer’s instructions. Complementary DNA (cDNA) was synthesized using the SweScript RT I First Strand cDNA Synthesis Kit (Servicebio, Wuhan, China). Each qPCR was performed in a 384-well plate; all reactions contained 2× Fast SYBR Green qPCR Master Mix (Servicebio, Wuhan, China), cDNA, and 0.25 μM forward and reverse primers. Relative mRNA expression levels were calculated using the 2^−ΔΔCq^ method, and β-actin served as the internal control. The reaction protocol was as follows: 95 °C for 10 min, followed by 45 cycles of 95 °C for 15 s, 60 °C for 1 min, and 72 °C for 15 s. All assays were performed in triplicate. The primer sequences were as follows: IFN-γ: forward 5′-TATTCGGTAACTGACTTG-3′, reverse 5′-TAATCACATAGCCTTGC-3′; IL-6: forward 5′-GGAGACTTGCCTGGTGAA-3′, reverse 5′-GCATTTGTGGTTGGGTCA-3′; IL-10: forward 5′-GGAGGACTTTAAGGGTTA-3′, reverse 5′-GTAGGCTTCTATGTAGTTGAT-3′; and β-actin: forward 5′-TCCCTGGAGAAGAGCTACGA-3′, reverse 5′-TGAAGGTAGTTTCGTGGATGC-3′.

### 2.10. Quantitative Analysis of Immunomodulatory Factors in Aqueous Humor by Enzyme-Linked Immunosorbent Assay (ELISA)

Aqueous humor samples were collected from the control group (*n* = 20) and the diabetic cataract group (*n* = 20). Levels of IFN-γ, IL-6, and IL-10 were measured using an ELISA kit (mlbio, Shanghai, China). Each sample was analyzed independently, and all procedures were performed in accordance with the manufacturer’s protocol. For sample preparation, aqueous humor samples stored at −80 °C were thawed at room temperature and centrifuged at 704 g for 30 min. Standard and sample wells were then prepared.

Fifty microliters of the standard solution was added to each standard well. For sample wells, 40 μL of the sample diluent and 10 μL of the sample solution (equivalent to a fivefold dilution) were added; the mixtures were gently shaken. Except for blank wells, 100 μL of the enzyme-labeled reagent was added to each well. The plates were sealed with a membrane and incubated at 37 °C for 60 min. After incubation, the plates were washed with a X1 wash solution for 30 s, the liquid was discarded, and the plates were dried. This washing step was repeated five times. Subsequently, 50 μL of developer A and 50 μL of developer B were added to each well; the plates were gently shaken and incubated at 37 °C for 15 min in the dark. The reaction was terminated by adding 50 μL of stop solution to each well. The absorbance (optical density) was measured at 450 nm within 15 min. The linear regression equation for the standard curve was generated from the standard concentrations and their corresponding optical density values. Optical density values for the samples were entered into this equation to calculate sample concentrations, which were then multiplied by a dilution factor of 5 to determine the actual concentrations.

### 2.11. Statistical Analysis

All data were statistically analyzed using GraphPad Prism (version 9.5) and R (version 4.0.2). The Shapiro–Wilk test was used to assess the normality of the data distribution. Data that conformed to a normal distribution were analyzed using unpaired *t*-tests, while the non-conforming data were analyzed using Wilcoxon rank-sum tests. Categorical data were analyzed using Fisher’s exact test. Unpaired *t*-tests and Fisher’s exact test were employed for statistical analysis. A *p*-value < 0.05 was considered statistically significant.

## 3. Results

### 3.1. Transcriptomic Analysis of PBMCs Indicates Immune Cell Migration

**Figure 1:**
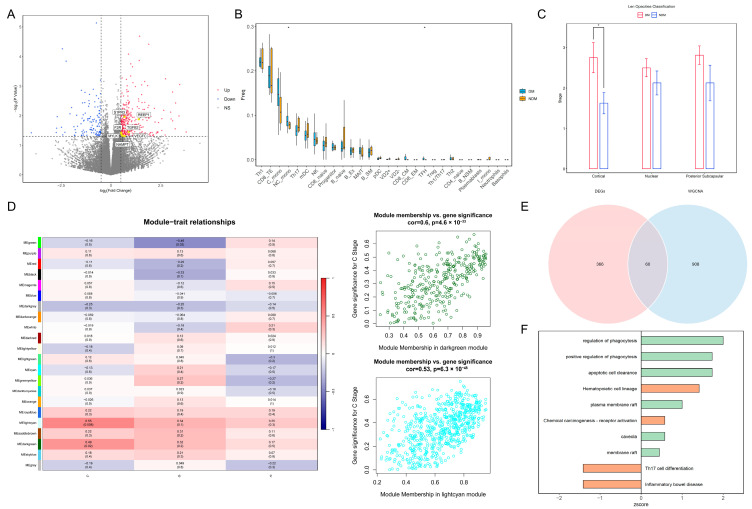
Transcriptomic analysis of PBMCs reveals changes in the peripheral blood immune landscape under diabetic conditions: (**A**) Volcano plot of DEGs. Yellow points indicate marker genes associated with vascular barrier disruption. (**B**) Box plot of differential immune cell proportions between the DM and NDM groups. * *p* < 0.05 (**C**) Bar plot of cataract opacity scores between the two groups. * *p* < 0.05. (**D**) Heatmap of correlations between WGCNA modules and clinical phenotypes. Dark-green and light-cyan modules are significantly associated with cortical opacity. C: Cortical; N: Nuclear; P: Posterior Subcapsular. (**E**) Venn diagram of genes overlapping between key modules and DEGs. (**F**) Bar plot of pathway enrichment for intersecting genes. Green indicates GO pathways, orange indicates KEGG pathways, and the x-axis represents pathway z-scores. PBMCs: peripheral blood mononuclear cells; DEGs: differentially expressed genes; WGCNA: weighted gene co-expression network analysis; GO: Gene Ontology; KEGG: Kyoto Encyclopedia of Genes and Genomes.

The statistical analysis of the clinical data for the two patient groups is presented in Table 1. The results of the normality tests are presented in Table S1. For data that follow a normal distribution, results are presented as the mean and standard deviation (SD), while the results for non-conforming data are presented as the median and range. There were significant differences between the two groups in HbA1c, GLU levels, BCVA, and cortical opacity. The differential expression analysis identified 429 genes that were significantly different between the groups. Among these, seven marker genes—transforming growth factor beta 2 (*TGFβ2*), coagulation factor II receptor (*F2R*), sphingosine-1-phosphate receptor 3 (*S1PR3*), receptor accessory protein 1 (*REEP1*), myosin light chain kinase (*MYLK*), vascular endothelial growth factor C (*VEGFC*), and nicotinamide phosphoribosyltransferase (*NAMPT*)—associated with the blood–aqueous barrier (BAB), endothelial dysfunction, and monocyte chemotaxis, were upregulated in the diabetic group (Figure 1A). The immune infiltration analysis revealed increased abundances of non-classical monocytes and T follicular helper cells within the peripheral blood of patients in the diabetes group (Figure 1B). A statistically significant difference in cortical cataract opacity was observed between the two groups (*p* = 0.022) (Figure 1C). Therefore, we selected modules significantly associated with cortical opacity in WGCNA; the genes in these modules were included in the further analyses (Figure 1D). Overlapping these module genes with the DEGs yielded 60 intersecting genes for the pathway enrichment analysis (Figure 1E). The results demonstrated that pathways related to phagocytosis and lipid rafts were significantly upregulated in the diabetic group, suggesting increased immune cell migration in the peripheral blood environment (Figure 1F). Based on these findings, we speculate that diabetes impairs vascular endothelial function, enabling immune cells in peripheral blood to breach the BAB and migrate into the lens.

### 3.2. Single-Cell Sequencing Analysis of PBMCs Reveals Immune Cell Regulation in Diabetes

**Figure 2:**
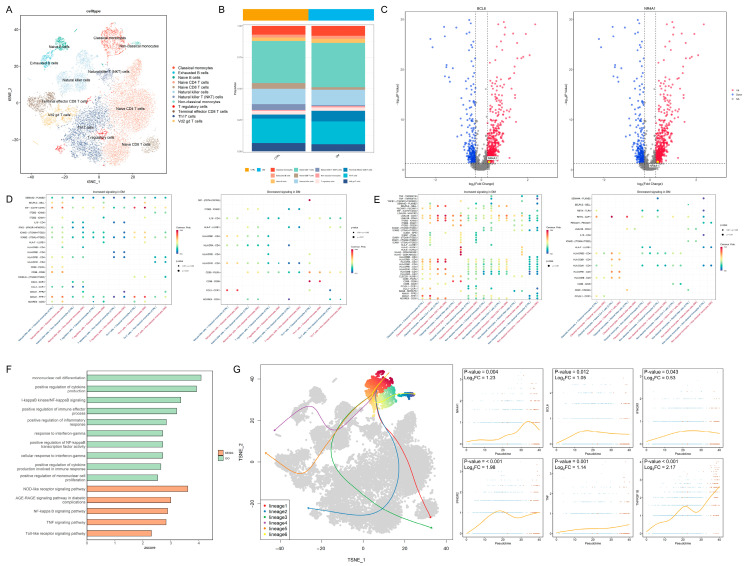
Intercellular regulatory relationships and developmental dynamics: (**A**) t-SNE plot of PBMCs divided into 12 cell types. (**B**) Cell abundance map showing increased proportions of both classical and non-classical monocytes in the peripheral blood of the diabetic group. (**C**) Volcano plot demonstrating upregulation of *BCL6* and *NR4A1* expression in classical monocytes of the DM group. (**D**) Signals received by monocytes from other cells: (**left**), upregulated signals in the DM group; (**right**), downregulated signals in the DM group. The red color in the x-axis label represents the control group, while the blue color represents the DM group. (**E**) Signals transmitted by monocytes to other cells: (**left**), upregulated signals in the DM group; (**right**), downregulated signals in the DM group. The red color in the x-axis label represents the control group, while the blue color represents the DM group. (**F**) GO and KEGG enrichment analysis of DEGs in classical monocytes, showing significant upregulation of inflammation-related pathways (z-score > 0, *p* < 0.05). (**G**) Potential developmental trajectories within the cell cluster (**left**) and expression of key genes over time during the transition from classical (Blue dots) to non-classical monocytes (Red dots) (**right**). t-SNE: t-distributed stochastic neighbor embedding; PBMCs: peripheral blood mononuclear cells; DM: diabetes mellitus; GO: Gene Ontology; and KEGG: Kyoto Encyclopedia of Genes and Genomes.

In total, 20,612 PBMCs were divided into 12 cell types (Figure 2A). The distribution of the marker gene expression is displayed in Figure S1A. The cell abundance plot reveals that the proportions of classical monocytes and non-classical monocytes are both higher in the DM group compared to the control group (Figure 2B). Transcription factors associated with non-classical monocytes, including B-cell lymphoma 6 protein (BCL6) and nuclear receptor subfamily 4 group A member 1 (NR4A1), exhibited higher expression levels in the classical monocytes of the diabetic group (Figure 2C). In terms of cell communication, the interferon-γ pathway received by classical monocytes and the tumor necrosis factor (TNF) pathway transmitted by non-classical monocytes were significantly upregulated in the diabetes group (Figure 2D,E). GO and KEGG enrichment analyses revealed that inflammation-related pathways, including IFN-γ and nuclear factor (NF)-κB signaling, were upregulated in the classical monocytes of the diabetic group (Figure 2F), indicating potentially enhanced monocyte-mediated inflammatory responses in diabetes. The developmental trajectory analysis demonstrated a progression from classical monocytes to non-classical monocytes; the transcription factor BCL6 and the inflammation-related proteins interferon-γ receptor 1, interferon-γ receptor 2, TNF, and TNF receptor superfamily member 1B (TNFRSF1B) exhibited an increased expression as differentiation advanced (Figure 2G). These findings suggest that monocytes in the diabetic state tend to transition toward non-classical monocytes, consistent with PBMC immune infiltration results (Figure 1B); this heightened inflammatory state may adversely affect lens cells.

### 3.3. Diabetes Induces Immune Cell Infiltration and Epithelial Apoptosis in the Lens

**Figure 3:**
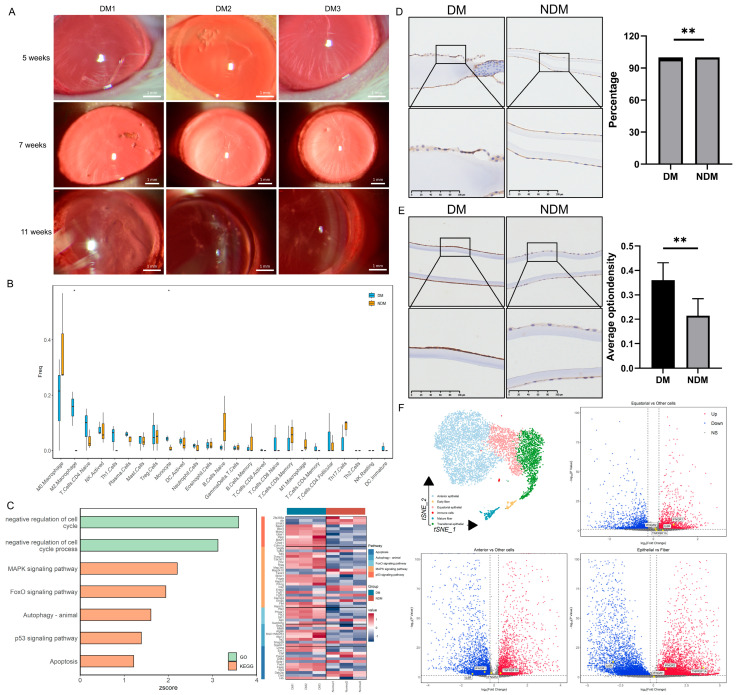
Transcriptomic analysis of lenses from diabetic cataract rats and immunohistochemical evaluation of the human lens capsule: (**A**) Establishment of the rat diabetic cataract model. (**B**) Box plot of immune cell proportions in the lens. * *p* < 0.05. (*n* = 6). (**C**) Bar chart of pathway enrichment analysis and heatmap of related gene expression levels. (**D**) Immunohistochemical staining of CD45 in human lens capsules. Quantitative analysis indicates enhanced immune cell infiltration in the DM group. ** *p* < 0.01. (*n* = 6). (**E**) Immunohistochemical staining of caspase-3 in human lens capsules. Quantitative analysis demonstrates increased apoptosis of lens epithelial cells in the DM group. ** *p* < 0.01. (*n* = 6). (**F**) t-SNE plot of lens cells divided into six cell types and a volcano plot of differentially expressed inflammatory factor receptors in lens epithelial cells compared with fiber cells. Red indicates upregulation; blue indicates downregulation. DM: diabetes mellitus; t-SNE: t-distributed stochastic neighbor embedding.

**Figure 4:**
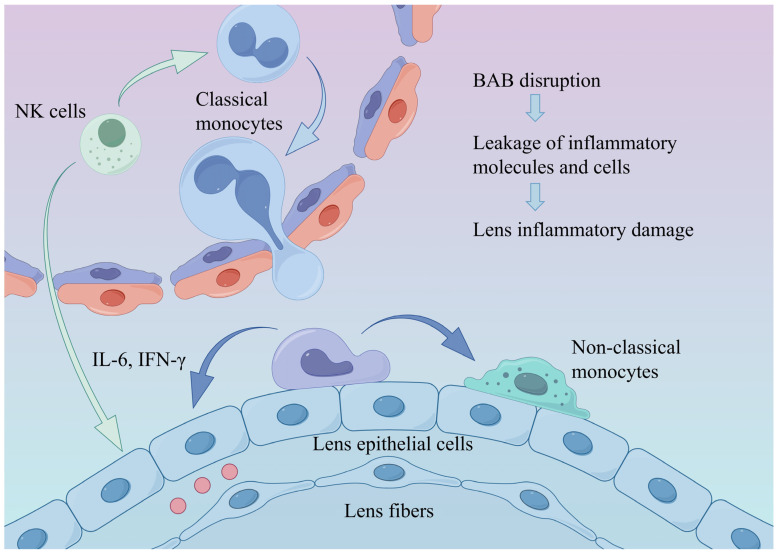
Alterations in the ocular immune microenvironment under diabetic conditions. Diabetes-induced hyperinflammation and disruption of the BAB promote monocyte recruitment and differentiation into non-classical monocytes, thereby exacerbating intraocular inflammation and leading to lens injury. BAB: blood–aqueous barrier.

In diabetic rats, cataracts were observed 5 weeks after modeling and significantly worsened by the 11th week (Figure 3A). The transcriptomic analysis revealed increased proportions of monocytes and M2 macrophages in the lenses of diabetic rats; activated natural killer (NK) cells and Th1 cells also exhibited an upward trend (Figure 3B). GO and KEGG analyses demonstrated a significant upregulation of the mitogen-activated protein kinase (MAPK) pathway, apoptosis, and negative cell cycle regulation in the diabetic group (z-score > 0) (Figure 3C). Similar findings were observed in human lens capsules. The number of CD45-positive cells was significantly higher in the diabetic group than in the non-diabetic group (*p* = 0.009) (Figure 3D). Additionally, the concentration of the apoptosis marker caspase-3 was significantly elevated in the diabetic group (*p* = 0.005) (Figure 3E). These results suggest that diabetes-induced apoptosis of lens epithelial cells is associated with immune regulation. The single-cell sequencing analysis of the lens produced similar results. Overall, 6616 lens cells were divided into 12 cell clusters and 6 cell types: anterior epithelial cells, equatorial epithelial cells, transitional epithelial cells, early fibers, late fibers, and immune cells. The distribution of the marker gene expression is presented in Figure S1B. The differential gene expression analysis across cell types revealed a higher expression of TNF-α and IFN-γ receptors in epithelial cells. Among these, the TNF-α receptor exhibited the highest expression in anterior epithelial cells, whereas the IFN-γ receptor was expressed most strongly in transitional epithelial cells. The IL-6 receptor was expressed at higher levels in fiber cells (Figure 3F). Based on the receptor distribution, we speculate that TNF-α signaling is the initiating factor for inflammation in anterior epithelial cells. IFN-γ signaling in transitional epithelial cells appears to induce apoptosis, preventing the replacement of damaged anterior epithelial cells, and IL-6 may affect lens fibers. Based on the integrated analysis, we propose that diabetes induces alterations in the immune microenvironment, as illustrated in Figure 4.

### 3.4. IL6 Levels Are Increased in Lens Epithelial Cells of the Diabetic Cataract Group

**Figure 5:**
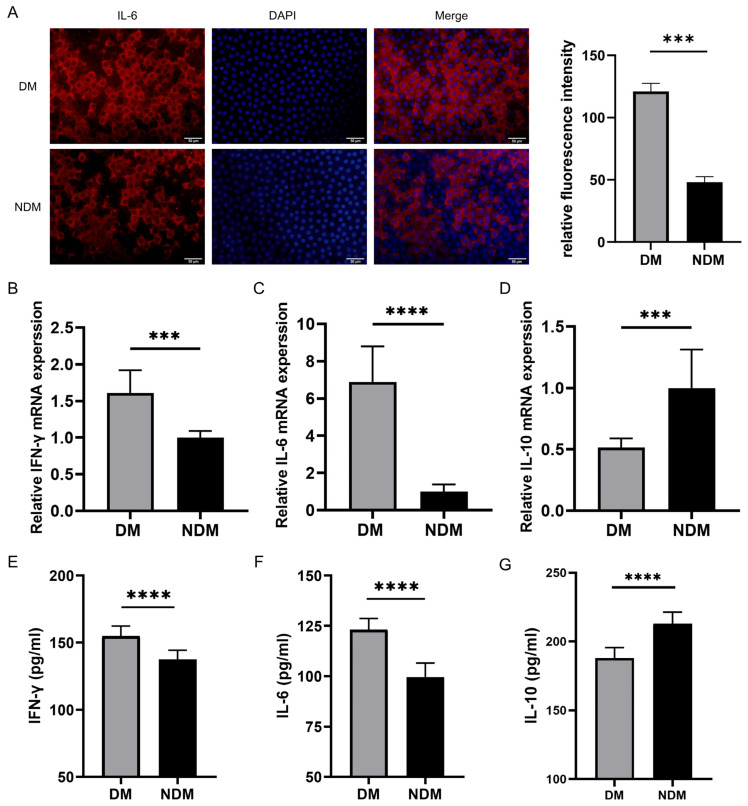
Experimental detection of IL-6, IFN-γ, and IL-10: (**A**) Representative immunofluorescence staining images of lens epithelial cells in the DM and NDM groups. Quantitative analysis demonstrated significantly higher IL-6 fluorescence intensity in the diabetic cataract group (*n* = 6). (**B**–**D**) qRT-PCR results showing increased expression of IFN-γ and IL-6 and decreased expression of IL-10 in the diabetic cataract group (*n* = 16). (**E**–**G**) ELISA results indicating elevated levels of IFN-γ and IL-6 and reduced levels of IL-10 in the aqueous humor of the diabetic cataract group. *** *p* < 0.001 and **** *p* < 0.0001 (*n* = 40). DM: diabetes mellitus.

The immunofluorescence staining of anterior lens capsules revealed a higher IL-6 expression in lens epithelial cells of the diabetic cataract (DM) group than in those of the non-diabetic cataract (NDM) group. As shown in Figure 5A, the IL-6 staining was more pronounced in the DM group, whereas it was weaker in the NDM group. A quantitative analysis confirmed a significant increase in IL-6 fluorescence intensity in the DM group (*p* < 0.001) (Figure 5A).

### 3.5. Immunomodulatory Detection via qRT-PCR and ELISA

The qRT-PCR analysis revealed elevated expression levels of IFN-γ and IL-6 in the diabetic cataract group compared with the simple cataract control group (all *p* < 0.0001), whereas the IL-10 expression was significantly decreased in the diabetic cataract group (*p* < 0.0001) (Figure 5B–D). These findings were validated by the ELISA (Figure 5E–G). In aqueous humor samples, IFN-γ and IL-6 levels were significantly increased, whereas IL-10 levels were reduced in the diabetic cataract group compared with the control group (all *p* < 0.0001).

## 4. Discussion

The transcriptomic analysis of peripheral blood from diabetic patients revealed alterations in BAB permeability and enhanced monocyte chemotaxis, along with an increased proportion of non-classical monocytes. Notably, a recent study reported that, under diabetic conditions, immune cell migration and infiltration into the lens occur even prior to the elevation of blood glucose levels, indicating that immune alterations play an indispensable role in the pathogenesis of diabetic cataracts [22]. These factors likely contribute to modifications of the ocular immune microenvironment. The eye is considered an immune-privileged organ, with the blood–ocular barrier effectively preventing the infiltration of external inflammatory mediators and inflammatory immune cells. However, recent studies have demonstrated that disruption of the BAB allows peripheral blood-derived inflammatory molecules and inflammatory cells to leak into the aqueous humor, leading to ocular tissue damage [23,24]. These inflammatory mediators and cells can subsequently activate intraocular cellular inflammatory responses, propagating inflammation and exacerbating the intraocular pro-inflammatory state [25].

Monocytes are considered classical inflammatory cells. In peripheral blood, monocytes predominantly exist as classical monocytes. Upon inflammation, they are recruited to and migrate into tissues, where they subsequently differentiate into non-classical monocytes [26]. Non-classical monocytes are closely associated with inflammation [27] and have been reported to increase in various autoimmune diseases and chronic inflammatory conditions [28,29,30]. The single-cell transcriptomic analysis revealed that the transcription factors BCL6 and NR4A1—required for the development of non-classical monocytes [31]—were strongly expressed in classical monocytes from the diabetic group. Similarly, bulk transcriptomic analysis revealed an increased *BCL6* expression. The cell trajectory analysis confirmed that BCL6 plays a critical role in the differentiation of classical monocytes into non-classical monocytes, and expression levels gradually increase during this transition. Previous studies have shown that *BCL6* expression is upregulated under conditions of oxidative stress, chronic hypoxia, and inflammation [32,33]. Pro-inflammatory cytokines, such as TNF-α, IL-6, and IL-8, can activate heat shock proteins, leading to the overexpression of *BCL6* [32,33]. Given that inflammation, hypoxia, and oxidative stress are common pathological features of diabetes, they may explain the elevated *BCL6* expression observed in this study [34,35].

The cell communication analysis revealed that classical monocytes in diabetic patients were characterized by higher levels of IFN-γ signaling from NK cells. Previous results suggest that IFN-γ signaling contributes to monocyte transformation; however, whether it facilitates the conversion of classical to non-classical monocytes remains unclear [36]. Non-classical monocytes in the diabetic group exhibited a distinct TNF signaling pathway, targeting classical monocytes and NK cells. Non-classical monocytes are considered the predominant producers of TNF-α [37], which promotes the recruitment and activation of other innate immune cells, such as NK cells [38], and enhances NK cell activity [39,40]. This mechanism may explain the increased IFN-γ levels observed in the diabetic group. Additionally, the TNF signaling pathway represents a canonical inflammatory pathway. Previous studies have established that TNF-α stimulates IL-6 production in monocytes by activating NF-κB [41,42]. This mechanism likely contributes to the elevated IL-6 levels detected in both the blood and aqueous humor of diabetic patients. Consistent with these findings, increased IL-6 expression in lens epithelial cells was confirmed through immunofluorescence staining.

The RNA sequencing analysis of rat lenses revealed a significant upregulation of MAPK and apoptosis pathways in diabetic rats—TNF-α and IL-6 were identified as key activators of MAPK signaling. A hyperglycemic environment is a significant risk factor for the onset and progression of diabetic cataracts [43,44,45]. Previous studies have reported excessive phosphorylation of p38 MAPK in the lenses of diabetic rat models [46]. The activation of the MAPK pathway indicates an increase in apoptosis, and damage to lens epithelial cells is considered a critical factor in cataract development and progression. Our single-cell transcriptome analysis of the lens demonstrated that epithelial cells broadly express IL-6 and IFN-γ receptors, providing a mechanistic basis for inflammatory injury in these cells. Experimental data further demonstrated elevated IL-6 levels in the lens capsules of diabetic patients, along with increased concentrations of IL-6 and IFN-γ in the aqueous humor relative to those in the normal control group. These findings suggest that the ocular environment in diabetic patients is characterized by a pro-inflammatory microenvironment, rendering lens epithelial cells more vulnerable to inflammatory damage. The single-cell transcriptome analysis also indicated immune cell infiltration in the lens. Although only a small number of immune cells were detected, this observation supports our hypothesis that the lens is not completely immune-privileged. Under high-inflammation conditions, resident immune cells within the lens may become activated and directly contribute to epithelial cell injury.

However, this study has some limitations. Based on the current findings, it remains unclear whether lens epithelial cell damage is caused directly by immune cell infiltration or indirectly by inflammatory mediators released from these cells. Additional experimental validation is required to clarify this mechanism. Our future research will focus on the intraocular immune microenvironment to provide more direct evidence of the relationship between immune modulation and cataract development.

In summary, we hypothesize that diabetes-induced alterations in the BAB and monocyte chemotaxis increase intraocular inflammation, which may directly or indirectly damage lens epithelial cells in diabetic patients. This immune-mediated injury likely contributes to elevated apoptosis and accelerates cataract progression.

## Figures and Tables

**Table 1:** Statistical results of patients’ baseline data.

Group	DM	NDM	*p*-Value
*n*	16	8	
Age, Years, Mean (SD ^1^)	66.75 (8.23)	68.63 (9.59)	0.645 ^2^
Sex (M/F)	6/10	1/7	0.352 ^3^
Duration, Years, Mean (SD)	13.88 (10.04)		
HbA1c, %, Median (Range)	7.2 (5.6–9.0)	5.75 (5.4–8.5)	0.027 ^4^
GLU, mmol/L, Median (Range)	7.8 (5.3–11.8)	5.75 (4.8–9.8)	0.025 ^4^
Eyes (OD/OS)	9/7	3/5	0.667 ^3^
UDVA, logMAR, Mean (SD)	1.34 (0.52)	1.00 (0.56)	0.176 ^2^
BCVA, logMAR, Median (Range)	1.2 (0.3–2.0)	0.46 (0.22–2.0)	0.021 ^4^
IOP, mmHg, Mean (SD)	15.83 (2.27)	15.61 (3.66)	0.884 ^2^
Len Opacity Classification			
Cortical, Median (Range)	3 (0–5)	1.5 (1–3)	0.025 ^4^
Nuclear, Median (Range)	3 (0–3)	2 (1–3)	0.101 ^4^
Posterior Subcapsular, Median (Range)	3 (1–4)	2 (0–4)	0.077 ^4^

^1^ Standard deviation. ^2^ Unpaired *t*-test. ^3^ Fisher’s exact test. ^4^ Wilcoxon rank-sum test.

## Data Availability

The RNA-seq files of rat lens and human PBMCs have been deposited in the GSA database under accession codes CRA019828 (https://ngdc.cncb.ac.cn/gsa/browse/CRA019828, accessed on 21 October 2024) and HRA009088 (https://ngdc.cncb.ac.cn/gsa-human/browse/HRA009088, accessed on 30 October 2024).

## Supplementary Materials

The following supporting information can be downloaded at https://www.mdpi.com/article/10.3390/biomedicines14020372/s1: Figure S1: Expression of marker genes. Table S1: Results of the normality tests.

## Abbreviations

The following abbreviations are used in this manuscript:

PBMCs	Peripheral Blood Mononuclear Cells
FC	Fold Change
DEGs	Differentially Expressed Genes
GO	Gene Ontology
KEGG	Kyoto Encyclopedia of Genes and Genomes
DM	Diabetes Mellitus
WGCNA	Weighted Gene Co-Expression Network Analysis
TOM	Topological Overlap Matrix
ME	Module Eigengene
MM	Module Membership
GS	Gene Significance
CCA	Canonical Correlation Analysis
MNNs	Mutual Nearest Neighbors
t-SNE	T-Distributed Stochastic Neighbor Embedding
PCs	Principal Components
AH	Aqueous Humor
SD	Standard Deviation
BAB	Blood–Aqueous Barrier
NK	Natural Killer
