# Supplementary material for: Inflammation-Mediated Immune Imbalance in the Pathogenesis of Diabetic Cataracts
Source: Biomedicines. 2026 Feb 5;14(2):372. doi: 10.3390/biomedicines14020372 (PMC12937691; doi:10.3390/biomedicines14020372)
Supplement: Supplementary file 1 [file biomedicines-14-00372-s001.zip › Supplementary Figure S1.pdf]

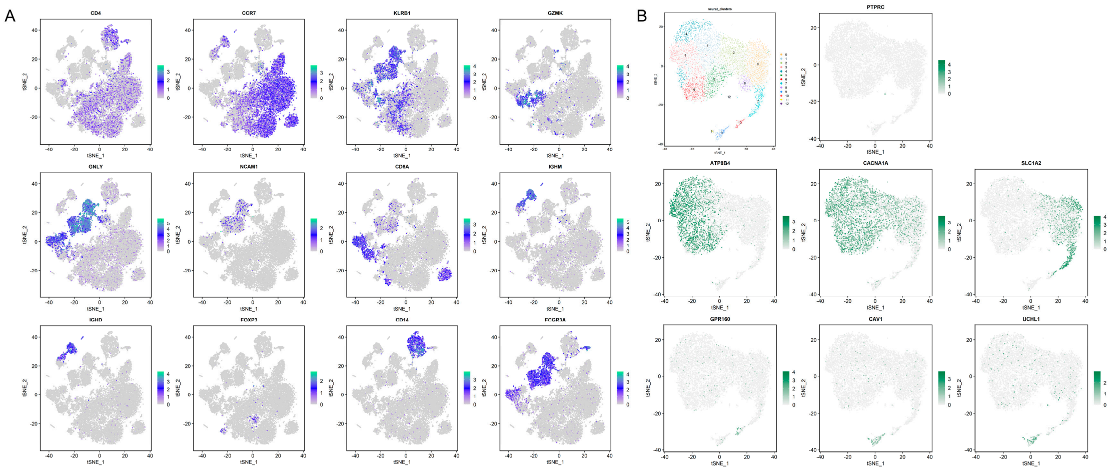

Figure S1. Expression of marker genes. (A) Expression distribution of marker genes used for PBMCs annotation. CD4: CD4+ T cell marker, CCR7: naïve T cell marker, CD161 (KLRB1): Th17 cell marker, GZMK: γδ T cell marker, GNLY: NK and NKT cell marker, CD56 (NCAM1): NK cell marker, CD8A: CD8+ T cell marker, IGHM: B cell marker, IGHD: naïve B cell marker, FOXP3: Treg cell marker, CD14: classical monocyte marker, CD16 (FCGR3A): non-classical monocyte marker. (B) Expression distribution of marker genes used for lens cells annotation. ATP8B4: anterior lens epithelial cell marker, CACNA1A: anterior and equatorial lens epithelial marker, SLC1A2: lens transitional epithelial marker, GPR160: early lens fiber marker, CAV1 and UCHL1: mature lens fiber markers.
